# IMPLEMENTATION OF THE ELDER MISTREATMENT EMERGENCY DEPARTMENT TOOLKIT AT HEYWOOD HOSPITAL

**DOI:** 10.1093/geroni/igac059.1594

**Published:** 2022-12-20

**Authors:** Carol-Lynne Papa, Randi Campetti, Ruthann Froberg, Kristin Lees Haggerty, Kim Dash

**Affiliations:** Heywood Hospital, Gardner, Massachusetts, United States; Education Development Center, Waltham, Massachusetts, United States; Education Development Center, Inc., Waltham, Massachusetts, United States; Education Development Center, Inc., Waltham, Massachusetts, United States; Education Development Center, Waltham, Massachusetts, United States

## Abstract

Beginning in 2016, Heywood Hospital, a 134-bed community hospital located in Central Massachusetts, partnered with the National Collaboratory to Address Elder Mistreatment to design and test a care model for identifying and responding to elder mistreatment (EM) in hospital emergency departments (ED). In February 2020, Heywood Hospital began implementing the care model known as the Elder Mistreatment Emergency Department (EMED) Toolkit as part of a five-site feasibility study. This presentation shares local results from the feasibility study and strategies for implementing the EMED Toolkit. 95% of Heywood staff participated in an online training program and, over 9 months, 4,588 (84%) of all older ED patients were screened for EM. Of these, 19 screened positive and 11 were reported to Adult Protective Services, a more than 7-fold (annualized) increase over the prior year. Most of the remaining positive cases were connected with community-based resources through Heywood’s Social Services Program.

